# Early Life Course Risk Factors for Childhood Obesity: The IDEFICS Case-Control Study

**DOI:** 10.1371/journal.pone.0086914

**Published:** 2014-02-13

**Authors:** Karin Bammann, Jenny Peplies, Stefaan De Henauw, Monica Hunsberger, Denes Molnar, Luis A. Moreno, Michael Tornaritis, Toomas Veidebaum, Wolfgang Ahrens, Alfonso Siani, on behalf of the IDEFICS consortium

**Affiliations:** 1 Institute for Public Health and Nursing Research (IPP), University of Bremen, Bremen, Germany; 2 BIPS Institute for Prevention Research and Epidemiology, Bremen, Germany; 3 Department of Public Health, Ghent University, Ghent, Belgium; 4 Department of Public Health and Community Medicine, University of Gothenburg, Gothenburg, Sweden; 5 Department of Pediatrics, University of Pécs, Pécs, Hungary; 6 Growth, Exercise, Nutrition and Development (GENUD) Research Group, University of Zaragoza, Zaragoza, Spain; 7 Research and Education Foundation of Child Health, Strovolos, Cyprus; 8 Department of Chronic Diseases, National Institute for Health Development, Tallinn, Estonia; 9 Unit of Epidemiology and Population Genetics, Institute of Food Sciences, National Research Council, Avellino, Italy; University of Missouri-Kansas City, United States of America

## Abstract

**Background:**

The early life course is assumed to be a critical phase for childhood obesity; however the significance of single factors and their interplay is not well studied in childhood populations.

**Objectives:**

The investigation of pre-, peri- and postpartum risk factors on the risk of obesity at age 2 to 9.

**Methods:**

A case-control study with 1,024 1∶1-matched case-control pairs was nested in the baseline survey (09/2007–05/2008) of the IDEFICS study, a population-based intervention study on childhood obesity carried out in 8 European countries in pre- and primary school settings. Conditional logistic regression was used for identification of risk factors.

**Results:**

For many of the investigated risk factors, we found a raw effect in our study. In multivariate models, we could establish an effect for gestational weight gain (adjusted OR = 1.02; 95%CI 1.00–1.04), smoking during pregnancy (adjusted OR = 1.48; 95%CI 1.08–2.01), Caesarian section (adjusted OR = 1.38; 95%CI 1.10–1.74), and breastfeeding 4 to 11 months (adjusted OR = 0.77; 95%CI 0.62–0.96). Birth weight was related to lean mass rather than to fat mass, the effect of smoking was found only in boys, but not in girls. After additional adjustment for parental BMI and parental educational status, only gestational weight gain remained statistically significant. Both, maternal as well as paternal BMI were the strongest risk factors in our study, and they confounded several of the investigated associations.

**Conclusions:**

Key risk factors of childhood obesity in our study are parental BMI and gestational weight gain; consequently prevention approaches should target not only children but also adults. The monitoring of gestational weight seems to be of particular importance for early prevention of childhood obesity.

## Introduction

Excess body fat is one of the major health concerns in childhood populations [1], [2]. Although the energy imbalance resulting from high energy intake and low energy expenditure can contribute to the development of overweight and obesity in children, there is no general consensus about its importance as the main driver of weight gain [3]. In recent years, factors early in the life course emerged as possible determinants of early overweight and obesity [4], [5]. These can lead to epigenetic changes which are associated with the programming of metabolic outcomes in the offspring. Different pre-, peri- and postnatal risk factors for childhood obesity have been investigated and there is an increasing need to disentangle the contribution of factors like maternal weight, gestational weight gain, glycemic control, smoking and alcohol use during pregnancy, birth weight, breast feeding on the adiposity of the offspring, for which partly inconsistent results have been obtained [5]. While high birth weight was consistently shown to be a risk factor for obesity, the situation is less clear with low birth weight that might or might not play a role in the development of subsequent overweight and obesity [6]. Likewise, the impact of breastfeeding on childhood obesity is still a matter of research [7]. Although a protective effect of breastfeeding for subsequent obesity of the offspring has been demonstrated in several studies, this result might at least partly be due to confounding: Amir and Donath show in their studies that maternal smoking and obesity lead to a lesser probability of breastfeeding [8], [9], both, maternal smoking and maternal obesity, being themselves strong risk factors for childhood overweight [5]. Correspondingly, it is suspected by some authors that the association of breastfeeding and childhood overweight is a statistical artifact rather than a true causal association [10].

In summary, there is still a considerable lack of research regarding the effect of early life course factors on the risk of childhood obesity.

The IDEFICS study is a large European multi-center intervention study on childhood obesity. Prior to the intervention phase, a baseline survey was conducted in the control and intervention regions to assess a wealth of factors related to diet- and lifestyle-related diseases in childhood. We used this data to set up a matched case-control study on obesity. The aim of the paper is to investigate the impact of pre-, peri- and postnatal risk factors on the subsequent risk of obesity within this case-control study.

## Materials and Methods

### Ethics Statement

We certify that all applicable institutional and governmental regulations concerning the ethical use of human volunteers were followed during this research, and that the IDEFICS project passed the Ethics Review process of the Sixth Framework Programme (FP6) of the European Commission. Ethical approval was obtained from the relevant local or national ethics committees by each of the eight study centers, namely from the Ethics Committee of the University Hospital Ghent (Belgium), the National Bioethics Committee of Cyprus (Cyprus), the Tallinn Medical Research Ethics Committee of the National Institutes for Health Development (Estonia), the Ethics Committee of the University Bremen (Germany), the Scientific and Research Ethics Committee of the Medical Research Council Budapest (Hungary), the Ethics Committee of the Health Office Avellino (Italy), the Ethics Committee for Clinical Research of Aragon (Spain), and the Regional Ethical Review Board of Gothenburg (Sweden). All parents or legal guardians of the participating children gave written informed consent to data collection, examinations, collection of samples, subsequent analysis and storage of personal data and collected samples. Additionally, each child gave oral consent after being orally informed about the modules by a study nurse immediately before every examination using a simplified text. This procedure was chosen due to the young age of the children. The oral consenting process was not further documented, but it was subject to central and local training and quality control procedures of the study. Study participants and their parents/legal guardians could consent to single components of the study while abstaining from others. All procedures were approved by the above-mentioned Ethics Committees.

### Study Sample

IDEFICS is a multi-center population-based intervention study on childhood obesity that is carried out in selected regions of 8 European countries comprising Belgium, Cyprus, Estonia, Germany, Hungary, Italy, Spain and Sweden. The study was set up in pre- and primary school settings in a control and an intervention region in each of these countries. Two major surveys (baseline and follow-up) were conducted in pre-schools and primary school classes (1^st^ and 2^nd^ grades at baseline). The baseline survey (September 2007–May 2008) reached a response proportion of 51% and included 16 220 children aged 2 to 9 years. The general design of the IDEFICS study has been described elsewhere [11]. A brief description of the study regions can be found in Bammann et al. [12].

Anthropometric measurements were done during a physical examination. Weight to the nearest 0.1 kg and foot-to-foot bioelectrical resistance in Ohm was measured using an electronic scale TANITA BC 420 SMA (TANITA Europe GmbH, Sindelfingen, Germany) with the children being in a fasting status and wearing only underwear. Standing height was measured with the children’s head in a Frankfort plane using a stadiometer SECA 225 (seca GmbH & Co. KG., Hamburg, Germany) to the nearest 0.1 cm. As in the weight measurement, the children were wearing only underwear, all hair ornaments were removed and all braids undone. Body mass index (BMI) was calculated as weight (kg) divided by height squared (m). For obtaining the International Task Force of Obesity (IOTF) category, BMI categories were interpolated for continuous age as proposed [13], [14]. Cubic splines were used for this interpolation. The resistance index (RI) was calculated as squared height (cm^2^) divided by resistance (Ohm). The RI was shown to be a good predictor for fat free mass in children [15].

Within the baseline survey, a self-administered questionnaire was filled in by the parents to gather information on the children’s behavior, parental attitudes and on the social microenvironment of the children (IDEFICS parental questionnaire). A further questionnaire on health-related and medical information was given to the parents in the course of the physical examination of the child (IDEFICS medical questionnaire). Questionnaires were developed in English, translated to the respective languages and back translated to English to minimize any heterogeneity due to translation problems. Different language versions were available in the centers, and help was offered to those parents who felt they were not able to fill in the questionnaire by themselves. For filling in the IDEFICS medical questionnaire, the help of medical personnel was offered directly at the survey sites.

Eligible for the IDEFICS case-control study on obesity were all children from the baseline survey for whom a basic set of anthropometric indicators and pregnancy information was present (N = 16,113). From these children, all children aged 4 to 8 years that met the IOTF criteria for childhood obesity were identified as cases (N = 1,024). Controls were selected from the group of IOTF normal weighted children (N = 11,193) and matched 1∶1 by study center, sex and age (sliding window +/−0.5 years) to the cases. For all cases, controls with identical sex and study center and a minimal distance with respect to age were selected. The decision for a 1∶1 matched design was based on a sample size calculation. In our study, risk factors with prevalences among controls of at least 10% leading to odds ratios of 1.6 and more can be detected with a power of more than 90% (two-sided, p = 0.05).

### Investigated Risk Factors

The investigated risk factors directly related to the child can be grouped into prepartum, peripartum, and postpartum factors (see Table 1). Further family-related risk factors included in the present analysis are familial clustering and parental social background. All information was assessed by the IDEFICS parental questionnaire, except for the occurrence of gestational diabetes that was taken from the IDEFICS medical questionnaire. All investigated risk factors are based on self-report.

**Table 1:** Risk factors investigated in the study.

Risk factor	Hypotheses
***Prepartum***	
Smoking during pregnancy	Own causal effect (risk factor) e.g. through fetal growth retardation and subsequent developmental adaptations [46], [47].
	Possible confounder: Parental energy intake [47], parental socioeconomic status [46].
Gestational weight gain	Own causal effect (risk factor) e.g. through shared genetic factors (on weight gain), shared environment (e.g. diet), fetal programming [48], [49].
	Possible confounder: Maternal BMI [49].
Gestational diabetes	Own causal effect (risk factor) e.g. through shared genetic factors, shared environment, fetal programming [50].
	Possible confounder: Maternal BMI.
***Peripartum***	
Birth weight	Own causal effect (risk factor) reflecting fetal growth, programming for lean mass and fat distribution [32].
	Possible artifact due to high correlation of BMI and lean body mass [32].
Caesarian section	Own causal effect (risk factor) e.g. through differences in colonizing bacteria species [34].
	Possible confounder: Maternal BMI, maternal smoking during pregnancy, breastfeeding [34].
***Postpartum***	
Breastfeeding (initiation and duration)	Own causal effect (protective factor) e.g. through nutritional programming [51], [52], moderation of genetic effects [53], reduced risk of overfeeding [54].
	Known confounder: parental obesity, maternal smoking during pregnancy, parental socioeconomic status; might completely remove the effect [55].
	Association possibly artificial due to lower breastfeeding success in obese and/or smoking mothers [10], [26].
Early introduction of solid foods	Own causal effect (risk factor) e.g. through nutritional programming [56].
	Possible confounder: Parental socioeconomic status [57], breastfeeding [58], [59].

Included prepartum factors were smoking during pregnancy, gestational weight gain and gestational diabetes. The questions relating to smoking during pregnancy and to gestational weight gain were posed to biological mothers, only. Regarding smoking during pregnancy, possible answer categories were “Never”, “Rarely, at maximum once a month”, “Several occasions a week” or “Daily”. We categorized “Rarely, at maximum once a month” or more often as smoking during pregnancy. Validity of maternal recall of smoking during pregnancy and of gestational weight gain is high even after several decades [16], [17].

Included peripartum factors were birth weight and information whether the child was delivered by Caesarian section. The validity of parental long-term recall of birth weight and of Caesarian sections is good to excellent [16]–[18], and is not related to time of recall since birth [19].

Included postpartum factors were duration of breastfeeding and introduction of solid foods. The early infant feeding of the child was assessed by a table indicating different types of feeding where starting and ending age could be given by the parents. The exact wording of the question was “What type of feeding with your child was used prior to being fully integrated into the usual household diet?”. Length of breastfeeding was calculated by subtracting starting age from ending age of breastfeeding, either exclusive or in combination with other types of feeding. The introduction of solid foods was assessed using the question “At what age did you first introduce …?” followed by a table with 5 different foods and the possibility of giving the time of introduction as age of the child in month. Early introduction of solid food was defined as introducing cereals, meat, vegetables or fruits at a child’s age before 4 months. Previous research has shown that the validity of maternal recall for duration of breastfeeding is very high, even after 10 years and more [16], [17], [20], [21]. Repeatability and validity for maternal recall of introduction of solid foods is shown to be higher than for introduction of fluids other than breast milk, but considerably lower than for breastfeeding with no clear direction of bias towards wrong recall of earlier or later dates [20], [22].

The familial clustering of overweight and obesity was assessed using self-reported parental BMI. The parental BMI was calculated as weight (kg)/squared height (m^2^).

The parental social background was described using the parental education and the age of mother at birth. For the analyses, these were transferred country-by-country to International Standard Classification of Education (ISCED) levels [12], [23] and the maximum ISCED level of both parents was calculated.

### Statistical Analyses

To evaluate the impact of a putative risk factor, conditional logistic regression models were fitted to the data. This allows an unbiased estimation of effects in matched case-control studies with regard to the matching variables. Within this approach, each matched set forms a stratum. In the case of 1∶1-matched pairs, the likelihood of being a case for N strata is then given by:
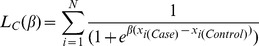



The estimation of the βs was done using Cox’ proportional hazard model with maximum likelihood estimation [24]. 95% confidence intervals (95% CI) were computed.

For all variables, the influence of the child’s sex and age group (< = 6 years, >6 years) on the raw odds ratio (OR) was tested using the Breslow-Day test for homogeneity of the odds ratios [25]. We found all OR to be homogenous regarding age group. OR that were found to be heterogeneous over sexes are reported in the text.

For each of the investigated factors, we built multivariate models adjusting for known or suspected confounders from the literature (cf. Table 1). Since many of the investigated risk factors are correlated, we finally built a multivariate model containing all factors that were shown to be influential in the analyses. We reported the Wald statistics to judge the relative importance of the single factors.

All statistical analyses were done with SAS 9.2 (SAS Institute, Cary (NC), USA).

Raw ORs for parental BMI and parental ISCED level can be found in the Appendix (Table S1).

## Results

A basic description of the study sample is displayed in Table 2. Roughly 50% of the case-control-pairs are male, 48% are 4–6 years, and 52% are 7–8 years old. The case-control pairs show large differences with respect to country of origin, ranging from 39.7% from Italy to 3.9 from Belgium and 2.7% from Sweden.

**Table 2:** Description of the study sample.

	Case-control pairs	
	N	%
***Sex***		
*Girls*	515	50.3
*Boys*	509	49.7
***Age***		
*4–6 years*	494	48.2
*7–8 years*	530	51.8
***Center***		
*Italy*	407	39.7
*Cyprus*	193	18.8
*Hungary*	135	13.2
*Germany*	81	7.9
*Spain*	77	7.5
*Estonia*	63	6.2
*Belgium*	40	3.9
*Sweden*	28	2.7
***Total***	**1,024**	**100**

The investigated early life course risk factors are shown in Table 3. Maternal smoking during pregnancy carried a 50% higher risk of the child being a case. Stratification by sex revealed that this elevated risk was present only in boys (OR = 2.15; 95% CI 1.43–3.23), but not in girls (OR = 1.13; 95% CI 0.78–1.62). The elevated obesity risk of maternal smoking during pregnancy remained practically unchanged when adjusting for parental BMI and parental educational level. Gestational weight gain showed a linear dose-response relationship mounting in a twofold risk of obesity if the mother gained 25 kg and more during pregnancy, which was not explained by maternal BMI. Also, gestational diabetes was associated with an excess risk of 32%. After adjustment for maternal BMI the elevated risk disappeared almost completely (OR = 1.05; 95%CI 0.57–1.94).

**Table 3:** IOTF obesity risk of early life course factors.

					Raw OR		OR adjusted for confounding factors		
	Controls		Cases		OR^a^	95% CI	OR^ab^	95% CI	Adjustment factors
*Maternal smoking during pregnancy*	N	%	N	%					Maternal BMI, parental educational level
*No*	857	88.0	809	83.1	1.00	–	1.00	–	
*Yes*	117	12.0	165	16.9	**1.52**	1.16–1.98	**1.50**	1.09–2.06	
*Gestational weight gain in kg*	N	%	N	%					Maternal BMI
*<10*	179	20.7	160	18.5	0.85	0.65–1.13	0.81	0.59–1.11	
*10–<15*	434	50.1	414	47.8	1.00	–	1.00	–	
*15–<25*	225	26.0	245	28.3	1.07	0.84–1.36	1.03	0.79–1.35	
*> = 25*	28	3.2	47	5.4	**2.00**	1.16–3.45	**2.11**	1.14–3.89	
*Gestational diabetes*	N	%	N	%					Maternal BMI
*No*	999	97.6	991	96.8	1.00	–	1.00	–	
*Yes*	25	2.4	33	3.2	1.32	0.79–2.22	1.05	0.57–1.94	
*Birth weight in kg*	N	%	N	%					Resistance index of the child
*<2.5*	73	7.6	44	4.5	**0.58**	0.39–0.86	1.00	0.55–1.82	
*2.5–4*	836	86.8	830	85.3	1.00	–	1.00	–	
*>4*	54	5.6	99	10.2	**1.83**	1.26–2.65	0.99	0.57–1.71	
*Caesarian section*	N	%	N	%					Maternal BMI, gestational weight gain, initiation of breastfeeding
*No*	662	70.4	596	62.7	1.00	–	1.00	–	
*Yes*	279	29.6	355	37.3	**1.43**	1.17–1.75	1.27	0.99–1.64	
*Breastfeeding*	N	%	N	%					Maternal BMI, parental educational level, maternal smoking during pregnancy
*No*	252	24.6	310	30.3	1.00	–	1.00	–	
*Yes*	772	75.4	714	69.7	**0.75**	0.61–0.91	0.91	0.70–1.18	
*Breastfeeding duration in months*	N	%	N	%					Maternal BMI, parental educational level, maternal smoking during pregnancy
*0*	252	24.6	310	30.3	1.00	–	1.00	–	
*>0–3*	171	16.7	181	17.7	0.86	0.66–1.12	1.03	0.73–1.44	
*4–6*	311	30.4	276	27.0	**0.71**	0.56–0.90	0.89	0.66–1.21	
*7–11*	218	21.3	170	16.6	**0.62**	0.47–0.81	**0.70**	0.49–0.99	
*> = 12*	72	7.0	87	8.5	0.93	0.65–1.35	1.25	0.80–1.94	
*Early introduction of solid foods*	N	%	N	%					Parental educational level, duration of breastfeeding
*No*	955	93.3	931	90.9	1.00	–	1.00	–	
*Yes*	69	6.7	93	9.1	**1.43**	1.02–2.01	1.33	0.93–1.90	

a Analyses were matched on sex, age and country.

b Analyses were additionally adjusted for putative confounders (see last column).

Matched odds ratios (OR) and 95% confidence intervals (95% CI): OR with p<0.05 are printed in bold.

The matched raw odds ratios for birth weight show a clear linear dose-response relationship with childhood obesity (OR = 0.58; 95%CI 0.39–0.86 for a birth weight <2.5 kg up to OR = 1.83; 95%CI 1.26–2.65 for a birth weight of >4 kg). However, when we adjusted for the RI as an indicator of fat free mass of the child, the effect of birth weight on the subsequent obesity risk disappeared completely. Being born via Caesarian section carried a higher obesity risk in our study (OR = 1.43; 95%CI 1.17–1.75). After adjustment for maternal BMI, maternal weight gain during pregnancy and initiation of breastfeeding, this risk was reduced and no longer statistically significant (OR = 1.27; 95%CI 0.99–1.64). The initiation of any breastfeeding carries an unadjusted OR of 0.75 (95%CI 0.61–0.91), and is thus protective against subsequent obesity in childhood. However, parental educational level, maternal BMI and maternal smoking during pregnancy almost completely explained this effect; the adjusted OR is near to unity (OR = 0.91; 95%CI 0.70–1.18). Likewise, the duration of breastfeeding shows unadjusted a u-shaped relationship to obesity with a protective effect only for the two middle categories (4–6 months, 7–11 months of non-exclusive breastfeeding). After adjustment for the afore-mentioned confounders, only 7–11 moths of non-exclusive breast-feeding remained statistically significant protective (OR = 0.70; 95%CI 0.49–0.99). Early introduction of solid foods shows an unadjusted OR of 1.43 (95% CI 1.02–2.01) that is reduced to a non-significant 1.33 (95% CI 0.93–1.90) after adjustment for parental educational level and initiation of breast-feeding.

To assess the combined effect of all investigated factors, we built two multivariate models (Table 4). The first model included gestational weight gain in kg, and design variables for smoking during pregnancy, Caesarian section, early introduction of solid foods and breastfeeding 4 to 11 months. The second model additionally included the confounding factors parental BMI in kg/m^2^ and parental educational level (unadjusted matched OR of these confounders can be found in Table A of the appendix).

**Table 4:** Multivariate models for pre-, peri- and postpartum risk factors on IOTF obesity risk.

	Model I			Model II		
	OR^ab^	95% CI	Wald	OR^ab^	95% CI	Wald
*Gestational weight gain in kg*	**1.02**	1.00–1.04	3. 827	**1.04**	1.01–1.07	8.717
*Smoking during pregnancy*	**1.48**	1.08–2.01	6.102	1.43	0.94–2.16	2.771
*Caesarian section*	**1.38**	1.10–1.74	7.558	1.17	0.87–1.57	1.015
*Breastfeeding 4 to 11 months*	**0.77**	0.62–0.96	5.415	0.83	0.62–1.11	1.552
*Early introduction of solid foods*	1.12	0.75–1.68	0.315	1.23	0.71–2.12	0.528
*Maternal BMI*				**1.16**	1.11–1.20	56.858
*Paternal BMI*				**1.11**	1.07–1.16	27.017
*Parental* *educational level*				0.92	0.81–1.04	1.686

a Analyses were matched on sex, age and country.

b Analyses were additionally adjusted for all parameters in the respective column.

Matched odds ratios (OR), 95% confidence intervals (95% CI) and Wald-statistics (Wald): OR with p<0.05 are printed in bold.

In the overall group, statistically significant risk factors for obesity were Caesarian section (adjusted OR = 1.38; 95%CI 1.10–1.74), gestational weight gain in kg (adjusted OR = 1.02; 95%CI 1.00–1.04), maternal smoking during pregnancy (adjusted OR = 1.48; 95%CI 1.08–2.01) and breastfeeding 4 to 11 months (adjusted OR = 0.77; 95%CI 0.62–0.96). After additional adjustment for parental BMI and parental educational level, statistically significant factors were maternal BMI in kg/m^2^ (adjusted OR = 1.16; 95%CI 1.11–1.20), paternal BMI in kg/m^2^ (adjusted OR = 1.11; 95%CI 1.07–1.16), and gestational weight gain in kg (adjusted OR = 1.04; 95%CI 1.01–1.07).

## Discussion

This paper investigated the impact of early life course risk factors on the risk of subsequent childhood obesity. For many of the investigated risk factors, we found a raw effect in our study. Our results regarding maternal smoking are confirmed by literature. In studies where maternal smoking was assessed, this factor is quite consistently reported to be a marked risk factor [26]–[28]. Similarly, high gestational weight gain was previously shown to be a risk factor not only for overweight and obesity of the child [29], but also of the mother [30]. Sometimes this effect is attributed to the influence of maternal adiposity [5], however in our study, gestational weight gain showed an independent effect, also after control for parental BMI. We were well in accordance with the literature, regarding high birth weight which was also a risk factor in our study [6], [26], [31]. However, when we adjusted for fat-free mass, this effect was completely removed, corroborating the hypothesis that birth weight is mainly influencing lean body mass and not fat mass [32]. Since the BMI is correlated with fat mass and with fat-free mass, one could argue that adjusting for fat-free mass leads generally to over-adjustment. Since the difference of fat-free mass between normal-weight and obese children is much lower than that of fat mass [33], we argue that adjustment for fat-free mass should not be able to mask any true effect on fat mass. Moreover, we checked for each of the other investigated factors whether adjustment for fat-free mass influences the OR. This was, apart from the effect for birth weight, not the case.

Caesarian section is another putative risk factor for childhood obesity that came into focus rather recently [34]. We also did find an elevated risk for obesity in the offspring, also after controlling for gestational gain, maternal BMI and breastfeeding initiation. However, in the final multivariate model Caesarian section was not of particular importance, when judged by the Wald statistic, and no longer statistically significant. The impact breastfeeding exerts on overweight is controversial since it is influenced by several other risk factors, as e.g. socioeconomic status, maternal BMI and maternal smoking [35]. One of the rare RCTs showed no effects of feeding breast milk on the prevalence of obesity until the age of 15 [36], suggesting that the protective effects found in other studies were due to statistical artifacts. Similarly, the RCT of Kramer et al. on breastfeeding promotion in preterm babies in Belarus showed no difference regarding obesity prevalence later in life in the two arms of the study [37]. Many previous studies have investigated either ever versus never-breastfeeding, while fewer have studied the duration of breastfeeding. Moreover, duration of breast-feeding is frequently modeled as months of breastfeeding assuming thus a strictly linear effect without any upper limit. In our study, we categorized the duration of breastfeeding and found a u-shaped effect. Interestingly, a similar pattern was found in a cohort study in Brazil [38] and in a secondary analyses of several cohorts examining the risk of duration of breastfeeding on cardiovascular risk factors [39]. However in our study, the effects were no longer statistically significant when controlling for confounding factors. As many other studies [5], [40]–[42], we also found a strong impact of parental weight status on the risk of childhood obesity. In a full model that included all investigated early life course risk factors for which we could establish an effect, gestational weight gain, smoking during pregnancy, Caesarian section were found to be risk factors for later childhood obesity, and breastfeeding 4 to 11 months was found to be protective. However, after additional adjustment for parental BMI and parental educational status, only gestational weight gain remained statistically significant. Both, maternal as well as paternal BMI were the strongest risk factors in our study, and they confounded several of the investigated associations. The current study has several limitations. It has to be kept in mind that the IDEFICS regions are not representative of the respective countries of Europe, let alone of the European population as a whole. Moreover, due to the high number of Italian obese children in the IDEFICS baseline survey (and the low number in Sweden and Belgium), the case-control sample was highly unbalanced with regard to country. As country was a matching variable this should not bias the results. We did not test for different effects by country, as we did for sex and age group, since the sample size was too small for most countries. For most of the associations that are hypothesized to be of a physiologic nature, like e.g. the association between Caesarian section and obesity, generalizability should not be compromised by the choice of regions. However, the association of social factors, like parental education, with childhood obesity is strongly dependent on context and therefore of limited external validity [12].

The investigated risk factors all rely on parental self-report. The validity of parental recall of most of the investigated factors is quite high, and we did not find much evidence for differential misclassification, which means that recall bias leads to risk attenuation rather than to distorted results. Recall bias might play a role in the lacking effect for introduction of solid food, as this factors has probably lowest validity among all investigated factors. Parental BMI is calculated using self-reported height and weight. Self-reported BMI is known to underestimate the true BMI, especially in the obese [43]. Assuming a familial clustering of obesity this produces a likely differential misclassification for parental BMI in our cases and controls. This misclassification also leads to attenuated Odds Ratios for paternal and maternal BMI, since especially among cases the proportion of overweight and obese parents is underestimated as opposed to the control group.

Our results show that parental BMI is of particular importance for childhood obesity. The parental BMI is a factor or an indicator for very different causal pathways, belonging to categories of shared genetics, a shared obesogenic environment or the process known as early programming. Currently many obesity intervention programs target children. However, reducing the prevalence of parental overweight and obesity would not only help preventing childhood obesity, but would in general lead to an improved health not only of the children, but also of their parents. Beyond this, our results indicate that especially maternal weight gain should be monitored closely during pregnancy. The mechanisms and possible prevention targets of maternal diet [44], [45] and other social and behavioral factors before and during pregnancy as risk factors for parental BMI and high gestational weight gain as well as for obesity in the offspring should be the subject of further studies.

## Supporting Information

Table S1 Distribution of continuous variables in the study population.
(DOCX)
