# Supplementary material for: Early Life Course Risk Factors for Childhood Obesity: The IDEFICS Case-Control Study
Source: PLoS One. 2014 Feb 13;9(2):e86914. doi: 10.1371/journal.pone.0086914 (PMC3923715; doi:10.1371/journal.pone.0086914)
Supplement: Table S1 — Distribution of continuous variables in the study population. (DOCX) [file pone.0086914.s001.docx]

**Table S1 Distribution (means and standard deviations (SD)) of continuous variables in the study population**

|  | **Controls** | | **Cases** | |
| --- | --- | --- | --- | --- |
|  | Mean | SD | Mean | SD |
| *Age in years* | 6.8 | 1.3 | 6.8 | 1.3 |
| *Weight in kg* | 23.4 | 4.3 | 36.8 | 8.1 |
| *Height in cm* | 120.9 | 9.4 | 125.8 | 9.8 |
| *Maternal BMI in kg/m^2^* | 23.6 | 3.7 | 26.5 | 5.3 |
| *Paternal BMI in kg/m^2^* | 26.7 | 3.5 | 28.5 | 4.2 |
| *Gestational weight gain in kg* | 13.6 | 5.4 | 14.3 | 6.1 |
| *Birth weight in g* | 3256 | 548 | 3365 | 564 |

**Table B IOTF obesity risk of confounding factors**

Matched odds ratios (OR) and 95% confidence intervals (95% CI): OR with p<0.05 are printed in bold

|  | **Controls** | | **Cases** | | **OR**^a^ | **95% CI** |
| --- | --- | --- | --- | --- | --- | --- |
| *Maternal BMI* | N | % | N | % |  |  |
| *<=25 kg/m2* | 700 | 73.1 | 442 | 46.6 | 1.00 | - |
| *>25-<=30 kg/m2* | 187 | 19.5 | 273 | 28.8 | **2.41** | 1.89-3.07 |
| *>30 kg/m2* | 71 | 7.4 | 233 | 24.6 | **5.60** | 3.98-7.89 |
|  |  |  |  |  |  |  |
| *Paternal BMI* | N | % | N | % |  |  |
| *<=25 kg/m2* | 305 | 34.9 | 171 | 19.7 | 1.00 | - |
| *>25-<=30 kg/m2* | 435 | 49.8 | 418 | 48.2 | **1.76** | 1.35-2.29 |
| *>30 kg/m2* | 133 | 15.2 | 278 | 32.1 | **4.04** | 2.91-5.62 |
|  |  |  |  |  |  |  |
| *Parental ISCED level* | N | % | N | % |  |  |
| *<=2* | 133 | 13.5 | 195 | 19.9 | 1.00 | - |
| *3* | 384 | 38.9 | 391 | 39.9 | 0.81 | 0.63-1.04 |
| *4* | 120 | 12.2 | 131 | 13.4 | 0.84 | 0.59-1.21 |
| *>=5* | 349 | 35.4 | 263 | 26.9 | **0.54** | 0.43-0.76 |

^a^ Analyses were matched on sex, age and country
